# The improved assembly of 7DL chromosome provides insight into the structure and evolution of bread wheat

**DOI:** 10.1111/pbi.13240

**Published:** 2019-09-18

**Authors:** Kewei Feng, Licao Cui, Le Wang, Dai Shan, Wei Tong, Pingchuan Deng, Zhaogui Yan, Mengxing Wang, Haoshuang Zhan, Xiaotong Wu, Weiming He, Xianqiang Zhou, Jingjing Ji, Guiping Zhang, Long Mao, Miroslava Karafiátová, Hana Šimková, Jaroslav Doležel, Xianghong Du, Shancen Zhao, Ming‐Cheng Luo, Dejun Han, Chi Zhang, Zhensheng Kang, Rudi Appels, David Edwards, Xiaojun Nie, Song Weining

**Affiliations:** ^1^ State Key Laboratory of Crop Stress Biology in Arid Areas College of Agronomy and Yangling Branch of China Wheat Improvement Center Northwest A&F University Yangling Shaanxi China; ^2^ College of Bioscience and Engineering Jiangxi Agricultural University Nanchang Jiangxi China; ^3^ Department of Plant Sciences University of California Davis CA USA; ^4^ BGI Genomics BGI‐Shenzhen Shenzhen China; ^5^ College of Horticulture and Forestry Sciences/Hubei Engineering Technology Research Center for Forestry Information Huazhong Agricultural University Wuhan China; ^6^ Key Laboratory of Crop Gene Resources and Germplasm Enhancement Ministry of Agriculture The National Key Facility for Crop Gene Resources and Genetic Improvement Institute of Crop Sciences Chinese Academy of Agricultural Sciences Beijing China; ^7^ Centre of the Region Haná for Biotechnological and Agricultural Research Institute of Experimental Botany Olomouc Czech Republic; ^8^ BGI Institute of Applied Agriculture BGI‐Shenzhen Shenzhen China; ^9^ State Key Laboratory of Crop Stress Biology for Arid Areas College of Plant Protection Northwest A&F University Yangling Shaanxi China; ^10^ State Agriculture Biotechnology Centre School of Veterinary and Life Sciences Australia Export Grains Innovation Centre Murdoch University Perth WA Australia; ^11^ School of Biological Sciences and Institute of Agriculture The University of Western Australia Perth WA Australia

**Keywords:** wheat, 7DL chromosome arm, BAC by BAC, physical mapping, domestication, gene loss

## Abstract

Wheat is one of the most important staple crops worldwide and also an excellent model species for crop evolution and polyploidization studies. The breakthrough of sequencing the bread wheat genome and progenitor genomes lays the foundation to decipher the complexity of wheat origin and evolutionary process as well as the genetic consequences of polyploidization. In this study, we sequenced 3286 BACs from chromosome 7DL of bread wheat cv. Chinese Spring and integrated the unmapped contigs from IWGSC v1 and available PacBio sequences to close gaps present in the 7DL assembly. In total, 8043 out of 12 825 gaps, representing 3 491 264 bp, were closed. We then used the improved assembly of 7DL to perform comparative genomic analysis of bread wheat (Ta7DL) and its D donor, *Aegilops tauschii* (At7DL), to identify domestication signatures. Results showed a strong syntenic relationship between Ta7DL and At7DL, although some small rearrangements were detected at the distal regions. A total of 53 genes appear to be lost genes during wheat polyploidization, with 23% (12 genes) as RGA (disease resistance gene analogue). Furthermore, 86 positively selected genes (PSGs) were identified, considered to be domestication‐related candidates. Finally, overlapping of QTLs obtained from GWAS analysis and PSGs indicated that TraesCS7D02G321000 may be one of the domestication genes involved in grain morphology. This study provides comparative information on the sequence, structure and organization between bread wheat and *Ae. tauschii* from the perspective of the 7DL chromosome*,* which contribute to better understanding of the evolution of wheat, and supports wheat crop improvement.

## Introduction

Bread wheat (*Triticum aestivum* L.) is one of the most important staple crops worldwide, providing around 19% of the total calories for humankind (FAO, www.fao.org/faostat). With the global population continuing to grow and climate change negatively impacting agricultural productivity, more efficient and systematic approaches are urgently required to breed improved wheat cultivars with a stable yield and are well‐adapted to diverse environmental stresses. The genome sequence of bread wheat is ultimately needed to better interpret the genetic variation and regulatory processes underlying key traits and to support the development of more effective breeding strategies (IWGSC, 2018).

Derived from a spontaneous hybridization of diploid *Aegilops tauschii* (2*n* = 14; DD) with tetraploid wheat *Triticum turgidum* (2*n* = 4*x* = 28; AABB) around 10 000 years ago, bread wheat (2*n* = 6*x* = 42) is a young hexaploid species with an AABBDD genome composed of three different homoeologous diploid genomes derived from *Triticum uratu* (AA), an *Aegilops* species related to the Sitopsis section (presumably *Ae. speltoides*; BB) and *Aegilops tauschii* (DD; Dubcovsky and Dvorak, 2007; Pont *et al*., 2019; Ramírez‐González *et al*., 2018). The *Triticeae* genomes have a large number of repetitive sequences (IWGSC, 2018; Luo *et al*., 2017), while polyploidization led to a complex (three sets of chromosomes with highly similar gene content) genome of bread wheat, with a large total size (more than 17 Gb) and high proportion (80%) of repetitive sequences (Akpinar *et al*., 2018). These biological features make wheat genome analysis a major challenge. Extensive efforts have tried to solve the assembly problem by using different sequencing approaches (Berkman *et al*., 2011, 2012; Brenchley *et al*., 2012; Chapman *et al*., 2015; IWGSC, 2014). The chromosome‐based strategy can simplify wheat genomic analysis to a manageable size and avoid the complexity of working with three homoeologous subgenomes. Based on this approach, the International Wheat Genome Sequencing Consortium (IWGSC) achieved three milestones, namely BAC‐by‐BAC assembly of high‐quality pseudomolecule of chromosome 3B (Choulet *et al*., 2014), the chromosome‐based draft sequence (IWGSC, 2014) and the fully annotated reference sequence of the bread wheat genome (IWGSC, 2018). Furthermore, reference‐quality genome sequences of several wheat progenitors have also been produced, including *Triticum uratu* (Ling *et al*., 2018), wild emmer (Avni *et al*., 2017), durum wheat (Maccaferri *et al*., 2019) and *Aegilops tauschii* (Luo *et al*., 2017). The high quality of these genome assemblies holds the promise to decipher the complexity of wheat's origin and the genetic consequences of polyploidization in this important crop (He *et al*., 2019). Although the fully annotated reference sequence of the bread wheat genome IWGSC v1 provides a valuable resource for wheat research, it also contains many unknown sequences (Ns), gaps and chimeras. For instance, a total of 5.7 Mb of unknown sequences (Ns), representing 12 825 gaps, were found in 7DL. In this study, we performed gap closure to improve the assembly of 7DL by integrating the unmapped sequence of IWGSC V1 version (400 Mb), available PacBio sequence data, as well as the sequence 3286 BAC clones for 7DL. This improved assembly was then used to investigate the evolutionary differences between *Ae. tauschii* and the D genome of hexaploid wheat using 7DL. The aim of this study was to provide insights into the sequence, structure and gene organization differences between bread wheat and *Ae. tauschii* from the perspective of the 7DL chromosome arm, which will lead to get a better understanding of the formation and evolution of wheat, and also support wheat crop improvement.

## Results

### Sequencing, assembly and annotation of wheat 7DL

A 7DL BAC library was constructed from DNA of flow‐sorted 7DL chromosome arms and comprises 50 304 clones with an average insert size of 116 kb, representing 14.9‐fold coverage of the predicted size of 346 Mb of 7DL (Šimková *et al*., 2011). The physical map construction of the 7DL chromosome resulted in 1614 contigs with an N50 of 349 kb and 6125 singleton clones. A total of 4457 clones were selected as a minimal tilling path (MTP) of the physical map, covering around 92% of the 7DL chromosome (Table 1). The MTP clones and 3286 manually selected singleton BAC clones were sequenced individually using Illumina sequencing technology. Additionally, DNA prepared from flow‐sorted 7DL arms was sequenced by Illumina and PacBio technologies, resulting in 26.5 Gb short reads and 3.3 Gb long reads, respectively. All of these data except for 3286 manually selected singleton BAC clones were used to perform a hybrid assembly which was anchored using a genetic map to produce a reference sequence of 7DL in IWGSC v1 (IWGSC, 2018; Figure S1). Based on this sequence of 7DL, we further closed the gaps by using the 3286 manually selected singleton BAC clones, the unmapped sequence of IWGSC v1 version (400 Mb) as well as publicly available PacBio sequences of cv. Chinese Spring (Clavijo *et al*., 2017). After manual correction and confirmation, 443 superscaffolds with an N50 of 887.6 kb were obtained, and the resulting pseudomolecule of 7DL was 280 672 331 bp in length (Table 1). Comparison of this assembly with the 7DL assembly of IWGSC v1 showed that we have closed 8043 gaps, with a total length of 3 491 264 bp, indicating that 66% of the total gaps in 7DL of IWGSC v1 were filled, providing a more complete reference sequence for 7DL (Table 2). To validate the assembly, the available deletion bin‐mapped EST, the full‐length cDNA sequences and four completely sequenced MTP BACs (randomly selected) from the PacBio platform (100×) were used to validate the assembly. Results showed that all of matched sequences showed perfect identity (Tables S1, S2, Figure S2). In addition, 163 regions were randomly selected using Sanger sequencing, and 149 fragments were successfully sequenced, of which 147 completely matched the 7DL assembly (Figure S3).

**Table 1:** Sequence assembly of 7DL chromosome arm.

	7DL assembly	Number/length
MTP Assembly	Number of 7DL BAC clones	50 304
	Number of MTP BAC clones	4457
	Number of contigs	1614
	BAC clones in contigs	37 367
	Number of singletons	6125
	Average contig length (kb)	300
	Longest contig length (kb)	2796
	Contig N50 (kb)	349
	L50 (contig number)	353
	Total contig length (Mb)	485.53
Superscaffold	Max length (bp)	2 852 487
	N50 (bp)	887 593
	N90 (bp)	320 416
	Total length (bp)	280 672 331
	Scaffold Number	443

**Table 2:** The gap closing of 7DL.

	Number of gap closure	Length of gaps (bp)	Average length
Unmapped_region_V1	33	28 986	878
Singleton BAC_Sequence	3261	1 338 596	410
PacBio_7DL	6932	3 026 326	437
Total	8043	3 491 264	434
7DL Gaps	12 825	5 798 173	452

**Figure 1:**
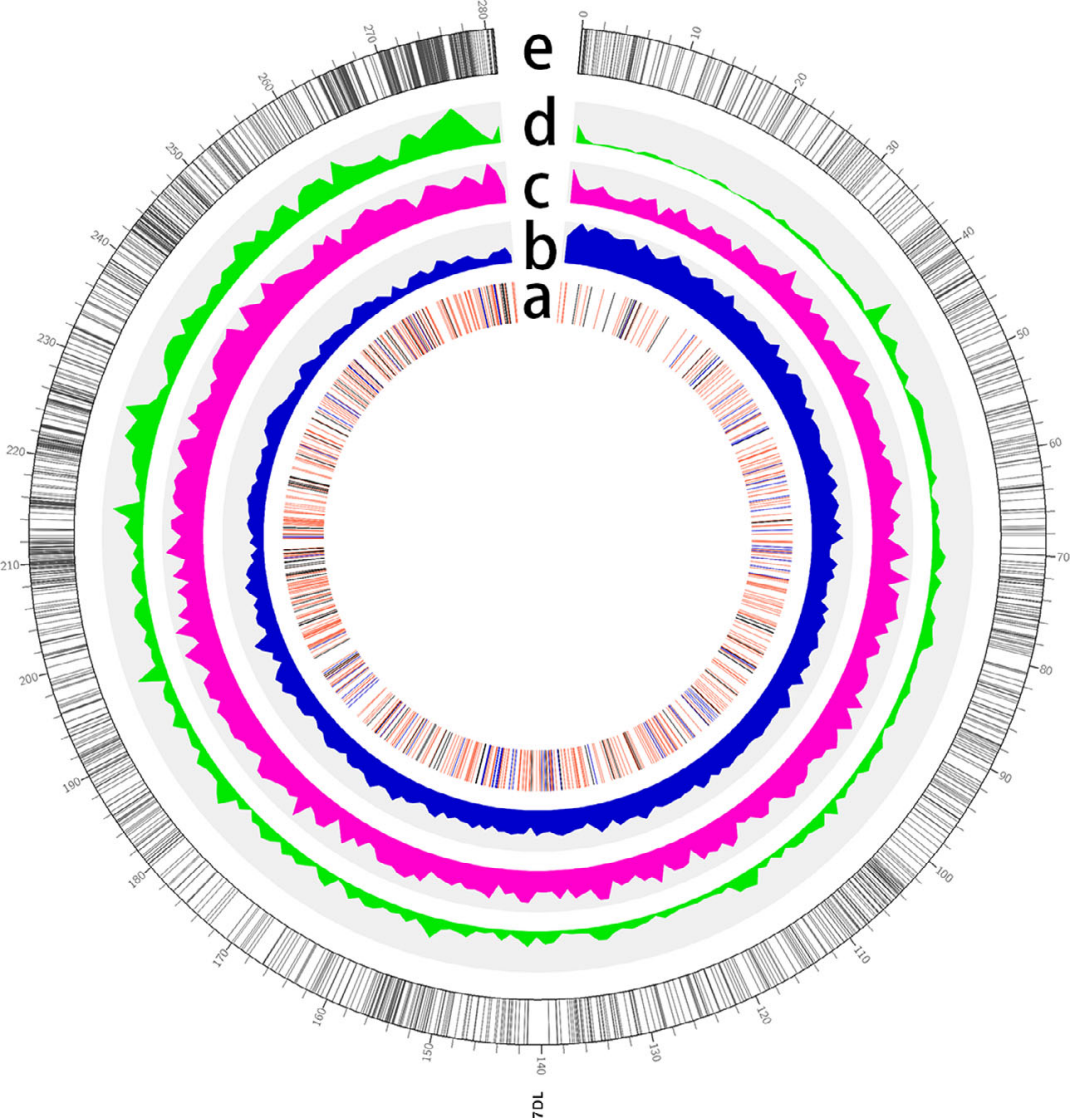
Genomic features of 7DL pseudomolecule. (a) Distribution of ncRNAs. (b) Density of *Gypsy*. (c) Density of *Copia*. (d) Density of high‐confidence genes in 7DL. (e): Distribution of genetic markers.

**Figure 2:**
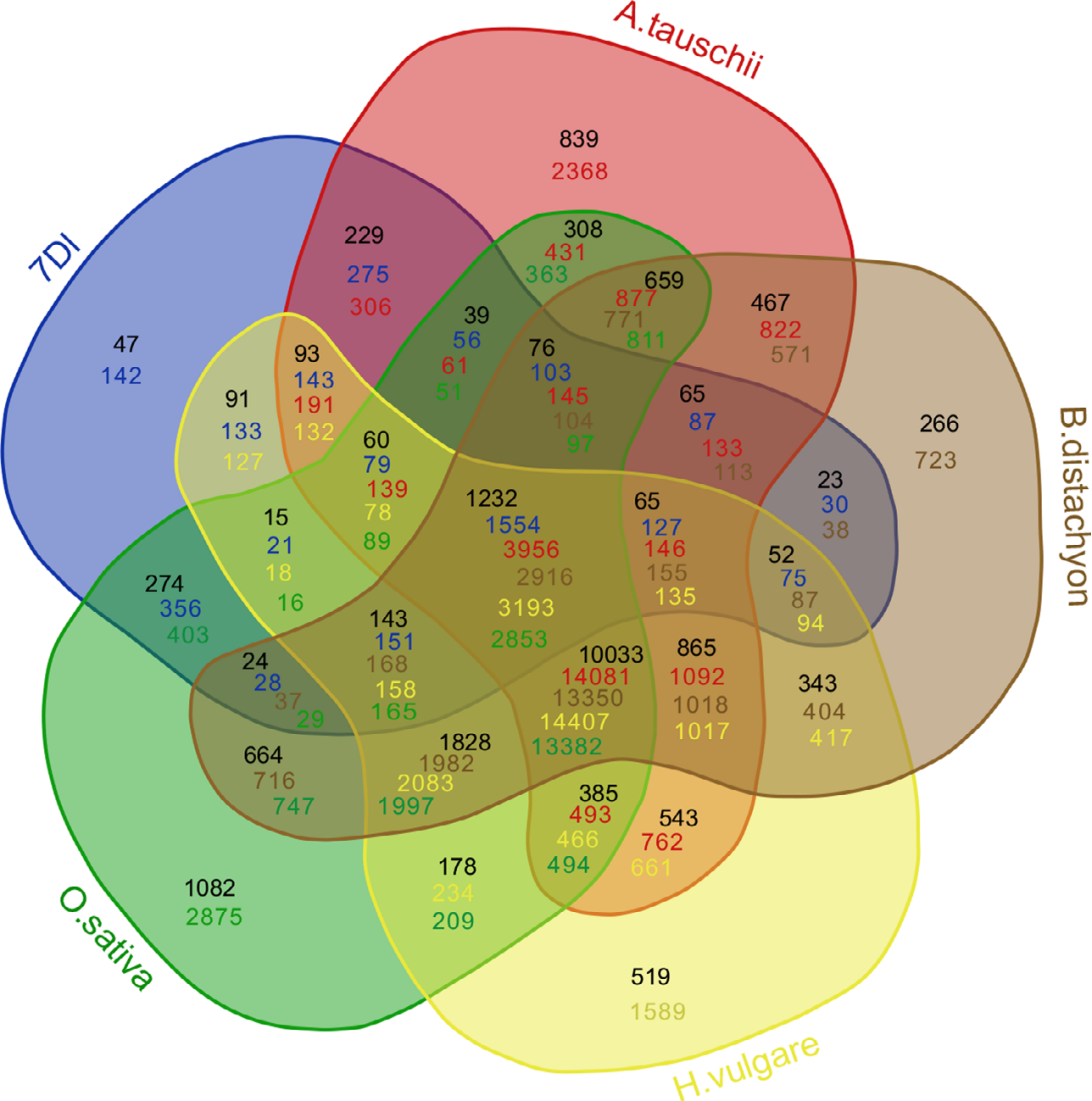
Comparison of gene families of wheat 7DL and homologous regions of related species of the grass family. Green: *Oryza sativa*; Brown: *Brachypodium distachyum*; Yellow: *Hordeum vulgare;* Blue: wheat 7DL.

A total of 3888 high‐confidence protein‐coding genes were predicted by combining *ab initio* and homology‐based methods, with an average length of 2210 bp and average exon number of 3.11 (Table 3; Figure S4), while 92 tRNAs, 73 rRNAs, 589 miRNAs, 76 snoRNAs and 838 lncRNAs were also identified (Table S3). The highest gene density observed along the pseudomolecule was 22 genes/Mb in the distal region, while the lowest density was 1.5 genes/Mb towards the centromeric region (Figure 1). Annotation analysis categorized 1423 genes into 44 GO terms (Table S4, Figure S5) and assigned 1954 genes to 21 KEGG pathways (Table S5, Figure S6). Gene family analysis together with homologous regions of four related species (*Ae. tauschii*,* Hordeum vulgare*,* Oryza sativa* and *Brachypodium distachyum*) indicated that the bread wheat 7DL chromosome shared most gene families with *Ae. tauschii*, which was consistent with their evolutionary relationship (Figure 2). Furthermore, 286 genes were identified as transcription factors, of which TRAF was the most abundant (Table S6). The expression of these high‐confidence genes was further validated by RNA‐Seq data (Table S7), of which 149 genes were found to be specifically expressed in the spike of the five tested tissues, and 322 genes are specifically expressed under cold stress among four stress conditions (Table S8, Figure S6).

**Table 3:** Annotation of 7DL pseudomolecule.

Type	Features	Size	Percentage
Protein‐coding genes	Total length (bps)	3 538 146	1.26
	GC content		53.49
	No. of genes	3888	
	Average size (bps) of coding sequences	910	
	Average no. of exon	3.11	
	Gene density (Mb)	14.5	
	No. of expressed Gene	3304	
Noncoding RNA genes	Total length (bps)	817 233	0.29
	No. of tRNA	92	
	No. of rRNA	73	
	No. of miRNA	589	
	No. of snRNA	76	
	No. of LncRNA	838	
Transposable elements (TEs)	Total length (bp)	221 818 927	78.97
	LTR/Gypsy	125 708 333	44.75
	LTR/Copia	66 615 535	23.72
	DNA/CACTA	32 232 071	11.47

Repetitive sequence analysis found that transposable elements accounted for 79% of the 7DL chromosome arm (Table S9), of which Gypsy is the most abundant (44.8%; Figure 1), followed by *Copia* (23.7%) and CACTA (11.5%) superfamilies. The density of the Gypsy superfamily gradually increased from the telomere towards the centromere, which exhibited a similar distribution to the total TE density, suggesting that the Gypsy LTR superfamily may be a major cause of variation in TE density along 7DL (Figure 1). Additionally, the insertion dates of the LTR retrotransposons were estimated to 0.25 MYA (Figure S7). In comparison with IWGSC v1.0, the gap‐closed 7DL sequences could improve the sequence completion of 7210 TE elements and 9 protein‐coding genes, which provides a more complete and correct annotation of wheat 7DL, in particular for TEs. More than 3000 improved TE elements belong to the Gypsy superfamily (Table S10).

### Comparative genome analysis between *T. aestium* (Ta7DL) and *Ae. tauschii* (At7DL)

**Figure 3:**
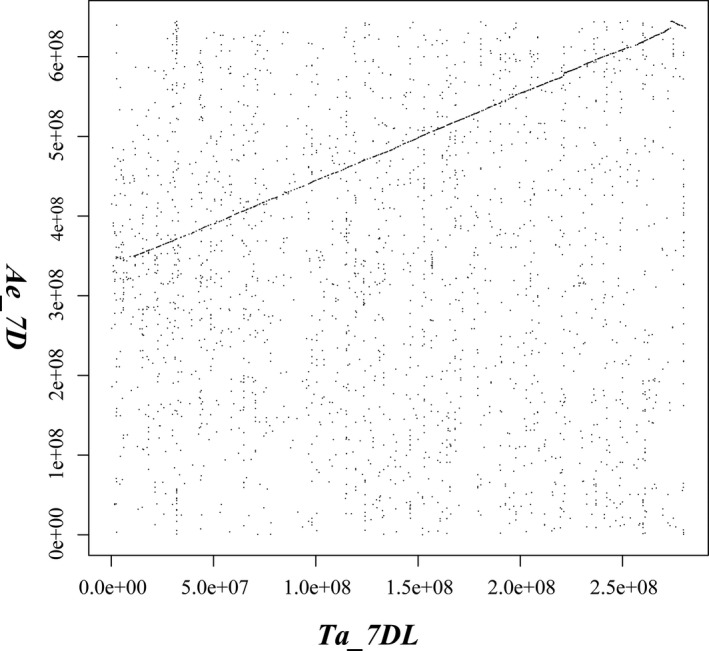
Dot plot of genome comparison between *Ta7DL* (horizontal axis) *and Ae7D* (vertical axis) chromosome.

Comparative genome analysis revealed a pronounced syntenic relationship (Figure 3) and gene order collinearity (Figure S8) between the 7DL arm of *T. aestium* (Ta7DL) and that of *Ae. tauschii* (At7DL). Only a small rearrangement was detected at a distal chromosome region, which is generally characterized by increased recombination frequency (Luo *et al*., 2017).

**Figure 4:**
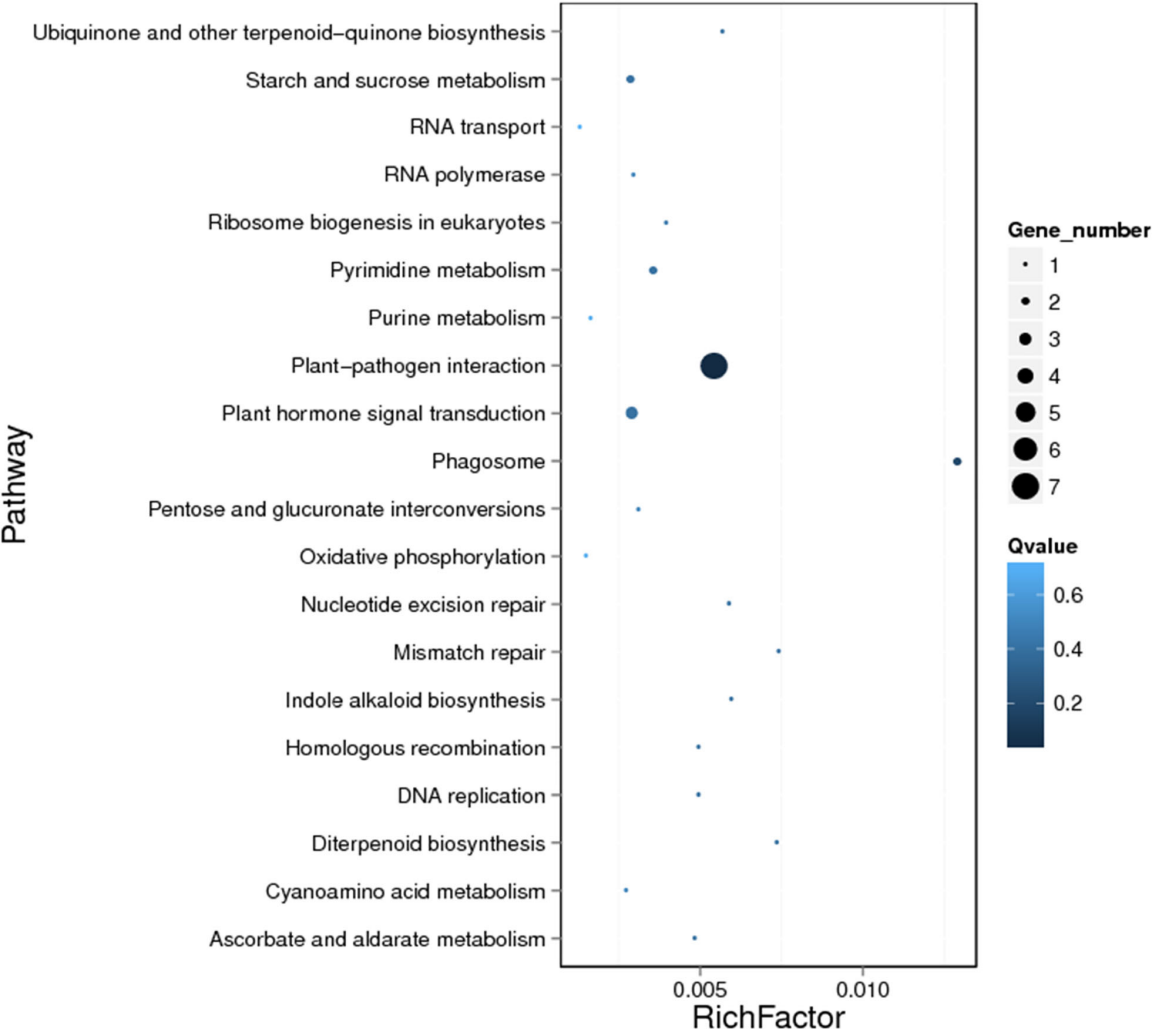
KEGG enrichment of lost genes of 7DL in *Ae. tauschii*.

Comparison of gene content and small‐scale molecular organization in bread wheat and *Ae. tauschii* showed that 113 genes on At7DL do not have orthologues on Ta7DL. However, 60 genes have orthologues in chromosomes 7A or 7B, or paralogues in one of the remaining chromosomes of wheat, while 53 genes are absent in wheat (Table S12). Most probably, these genes were lost during or after bread wheat formation. Interestingly, 12 (23%) out of the absent genes were identified as disease resistance gene analogue (RGA) and the proportion was significantly higher than that in *Ae. tauschii* (4.5%) or At7DL (4.7%; Fisher's exact test, *P* < 10^−5^; Table 4). Furthermore, functional enrichment showed that the lost genes were significantly enriched in plant–pathogen interaction pathway (ko04626, *P* < 0.001; Figure 4). The frequency of lost genes gradually increased from centromere to telomere (Figure S9), which is consistent with the gradient of recombination rate (Luo *et al*., 2017).

**Table 4:** Fisher's exact test of lost genes in 7DL.

	Number of RGAs in lost genes	Number of lost genes	Percentage
	12	53	23
	138	2917	4.70
Fisher's exact test	*P *=* *0.000004		
	1762	39 622	4.50
Fisher's exact test	*P *=* *0.0000035		

The difference between functional enrichment of genes on Ta7DL to the wheat whole genome as background and that of At7DL to the whole genome of *Ae. tauschii* was investigated to underline the role of 7DL's contribution to wheat formation. KEGG enrichment found that both of the chromosome arms were enriched in biosynthesis of zeatin (ko00908, *P* < 0.001), indole alkaloid (ko00901, *P* < 0.001) and ubiquinone and other terpenoid quinones (ko00130, *P* < 0.001; Figure S10). Additionally, genes on Ta7DL were enriched in energy metabolism‐related pathways such as oxidative phosphorylation (ko00190, *P* < 0.001) and photosynthesis (ko00195, *P* < 0.001), indicating that some divergences occurred after the formation of bread wheat.

**Figure 5:**
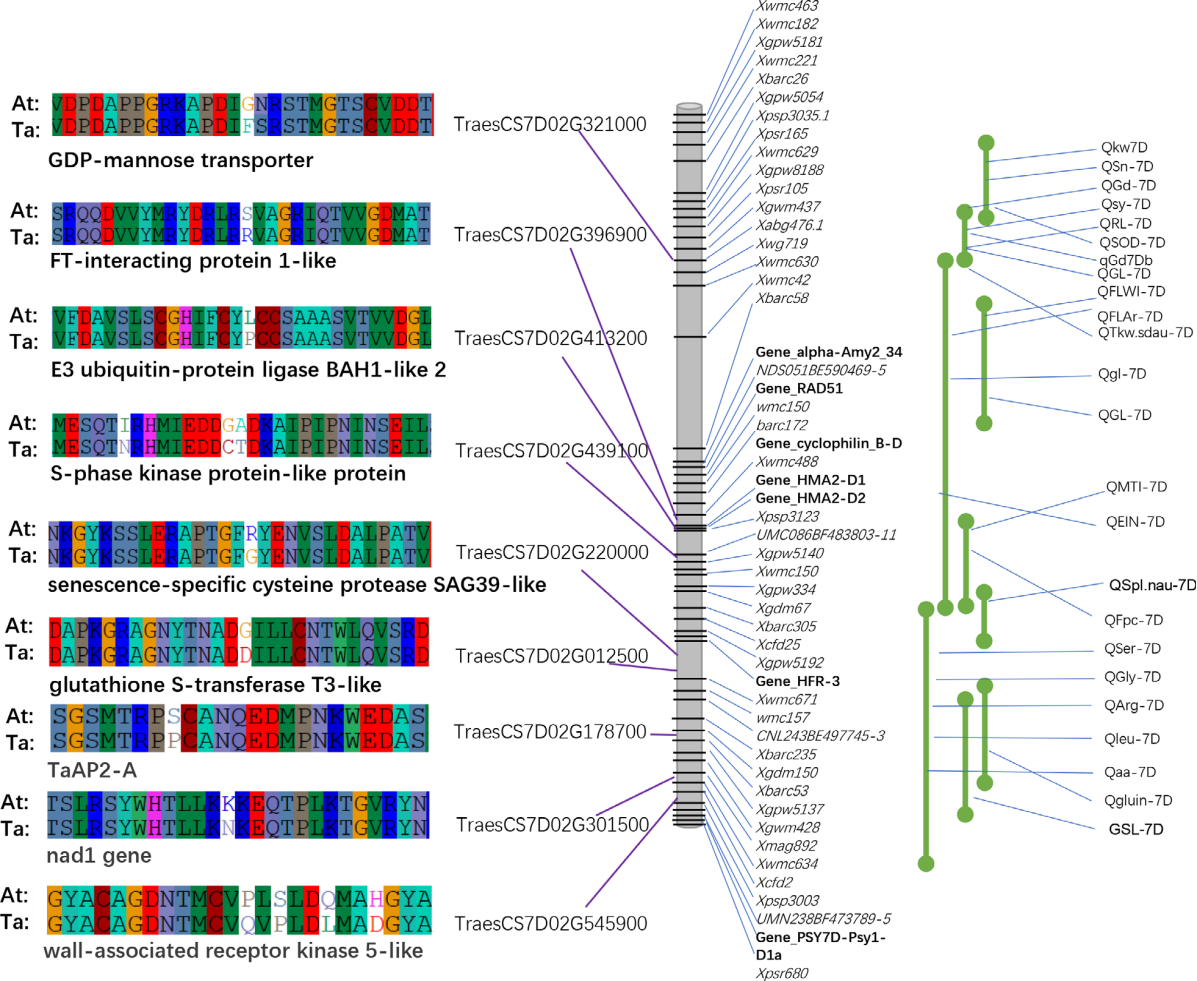
Location of putative QTLs and their close linked positively selective genes in 7DL pseudomolecule.

Orthologous gene pairs between Ta7DL and At7DL were identified, and dN, dS and dN/dS of each gene pair were calculated (Figure S11). A total of 86 genes were considered as positively selected genes (PSGs, dN/dS > 1), while 646 were negatively selected genes (NSGs, dN/dS < 1). Analysis showed that gene evolution rates correlated with gene GC content, length and expression level as well as codon bias characteristics (Figure S12). The comparison between PSGs and NSGs showed that PSGs have higher protein evolutionary rates, lower expression level and weaker codon bias than NSGs (Figure S13). Many important functional genes were found to be positively selected, such as the TaAP2‐A gene (TraesCS7D02G178700), FT‐interacting protein gene (TraesCS7D01G396900) and wall‐associated receptor kinase (TraesCS7D01G545900; Figure 5; Table S12). GO and KEGG analysis revealed that these PSGs were enriched in cytoskeletal protein binding term (GO: 0008092, *P* < 0.05) and the phenylpropanoid biosynthesis (ko00940, *P* < 0.05) pathway (Figure S14).

Previously reported molecular markers on 7DL were further aligned to the 7DL reference, and 46 markers corresponding to 37 QTLs were anchored (Figure 5; Table S11). The QTLs were related to important agronomic traits such as grain shape, thousand‐grain weight (QTkw.sdau‐7D and Qkw7D) and spike length (QSL‐7D and QSpl.nau‐7D), as well as related to amino acid content (QSer7D, QGly7D and QArg7D), providing valuable information for future fine mapping and gene cloning. Furthermore, nine PSGs were found to be closely linked with QTL markers in 7DL (Table S14). For example, a positively selected GDP‐mannose transporter gene (TraesCS7D02G321000) was found to be located in a 3‐Mb region flanked by markers for Xgwm437 and Xwmc630.1, which is linked to QTL loci QGd7D (grain diameter) and QGL7D (grain length), respectively. Extensive studies have demonstrated that grain morphologic traits are the most important domesticated traits in cereals (Meyer and Purugganan, 2013; Tian *et al*., 2015). To validate this QTL, we performed GWAS analysis of the wheat grain traits using 660K SNP array genotyping data of 310 accessions of bread wheat (including 24 landraces, 158 varieties and 128 breeding lines). The result of GWAS showed that the QTL signals associated with grain morphological traits such as grain diameter, grain area, grain length and width were mapped into the 3‐Mb region mentioned above on 7DL (Table S15), suggesting that the GDP‐mannose transporter gene (TraesCS7D02G321000) may be the candidate gene involved in controlling grain morphology.

## Discussion

At present, most assembled genomes contain gaps. It is still challenging to obtain complete genomes especially of the large complex genomes with high proportion of repetitive sequences such as bread wheat. Closing gaps after assembly would lead to more complete genomes, which benefits downstream genome analysis such as annotation and genotyping (Chu *et al*., 2019). The gap closure of 7DL improved the sequence quality of 7210 TE elements and nine protein‐coding genes (Table S10), which leads to better annotation, less genotyping error and easier identification of causal variation associated with traits in bread wheat.

The improved reference sequence of Ta7DL provided an opportunity to compare genome organization and gene content in bread wheat and *Ae. tauschii* from the perspective of this chromosome arm. Chromosomal rearrangements are a major driving force in shaping genome during evolution (Ma *et al*., 2015; Sankoff and Nadeau, 2003). The formation of hexaploid wheat through the hybridization of domesticated tetraploid wheat with *Ae. tauschii* was accompanied by a strong selection (Berkman *et al*., 2013). Our result showed only a small rearrangement was identified at distal chromosome regions (Figure 3) which are generally characterized by increased recombination frequency. It is known that the rate of recombination is higher in telomere regions and this may lead to translocations and inversions (Luo *et al*., 2017). The gene order collinearity between Ta7DL and At7DL was consistent with that of Ta5D and At5D (Akpinar *et al*., 2015). Because of shared ancestry, cereal genomes exhibit widespread collinearity, forming large ‘syntenic’ regions on chromosomes that carry orthologous genes. Gene order is largely collinear in grass species, which has proved helpful in both marker development and positional cloning (Helguera *et al*., 2015). Akpinar *et al*. (2018) compared the syntenic relationships and virtual gene orders between wild emmer wheat (*Triticum turgidum* ssp. *dicoccoides*) and grass genomes such as *Ae. tauschii*, and found several small‐scale evolutionary rearrangements. The similar observation in our research suggests that no large‐scale structural variation such as large tandem gene duplications, gene transpositions and chromosome rearrangements occurred in 7DL during the formation and domestication of hexaploid wheat. The result provides a basis for a systematic evaluation of gene presence or absence in the full spectrum of bread wheat and its close relatives, which could have significant implications in a wide array of fields to reveal evolutionary changes in the scope of chromosomes.

It is well known that fractionation following polyploidy generally causes the loss of sequences because of the combination of deletion and recombination of loci (Berkman *et al*., 2013). When the allotetraploid donor (AABB, *T. turgidum*) crossed with the D genome donor (*Ae. tauschii*) to form allohexaploid wheat (AABBDD), the D subgenome interacted with A and B subgenomes and some homoeologous sequences moved to homoeologous chromosomes or deleted due to recombination (Deng *et al*., 2014; Wang *et al*., 2016). It is reported that within a total of 39 622 genes, the number of RGA genes in *Ae. tauschii* was 1762 (Luo *et al*., 2017). Fisher's exact test indicated that the 53 lost genes in 7DL were significantly enriched for disease resistance genes (RGAs; *P* < 10^−5^; Table 4), and functional enrichment analysis also showed that these genes were significantly enriched in plant–pathogen interaction pathway related to environmental adaptation (ko04626, *P* < 0.001; Figure 4). The D genome donor *Ae. tauschii* has been reported representing a rich reservoir of biotic and abiotic stress tolerance for wheat stress improvement and adaption (Jia *et al*., 2013; Luo *et al*., 2012, 2017). The loss of environmental adaptation‐related genes in particular was probably the consequence of polyploidization and artificial selection (Reif *et al*., 2005; Xie and Nevo, 2008). Our result provides a case for detecting gene loss events between *Ae. tauschii* and bread wheat using the 7DL chromosome arm. Further studies on the presence or absence dynamics of stress‐related gene between wheat and its wild relatives could contribute to better understanding wheat evolution process and also provide the potential target genes for wheat genetic improvement. Additionally, the disease resistance genes in cultivated wheat are variable in different accessions (Montenegro *et al*., 2017), possibly reflecting differential loss following polyploidy. Genomes of different genotypes are needed to have a fuller picture of the gene loss in the hexaploid wheat gene pool during/after domestication. A similar gene loss pattern was also observed in another polyploidy crop, *Brassica napus*, with some agronomic trait‐related genes involved in flowering time, disease resistance and acyl lipid metabolism identified as absent due to homoeologous exchange (Hurgobin *et al*., 2018). The density of the lost genes (Figure S8) was lowest along the centromeric region and gradually increased towards the telomere region where the gradient of gene density and recombination rate also increases (Luo *et al*., 2017), suggesting that the gene loss may be related to ectopic recombination.

We found that genes on Ta7DL and At7DL were enriched in biosynthesis of zeatin (ko00908, *P* < 0.001), indole alkaloid (ko00901, *P* < 0.001) and ubiquinone and other terpenoid quinones (ko00130, *P* < 0.001; Figure S9). Zeatin is a member of the cytokinin family which involved in various processes of plant growth and development, such as regulating cell division and differentiation and increasing nutrient sink strength (Miyawaki *et al*., 2006; Werner *et al*., 2003). Indole alkaloids belong to the secondary metabolites, are important factors of plant resistance against microbial diseases and insects, and serve allelochemical functions (Grün *et al*., 2005). Ubiquinone carries electrons, acting as an energy carrier, and possesses antioxidant function (Cheng *et al*., 2003). Both Ta7DL and At7DL are enriched in such three related pathways, indicating 7DL chromosome plays quite similar role involved in plant growth, defence and energy conduction in bread wheat and *Ae. tauschii*. In addition, genes of Ta7DL were also enriched in energy metabolism‐related pathways such as oxidative phosphorylation (ko00190, *P* < 0.001) and photosynthesis (ko00195, *P* < 0.001), indicating some functional divergence has occurred between these homoeologous chromosome arms after the polyploidization. Akpinar *et al*. (2015) compared the gene enrichment difference of chromosome 5D between *Ae. tauschii* and *T. aestivum* and found that the Ta5D chromosome encodes a wider variety of genes related to the photosynthetic machinery and energy metabolism than that of the Aegilops 5D chromosome. Brenchley *et al*. (2012) pointed out that genes involved in energy harvesting, metabolism and growth might be associated with crop productivity. Both the 7DL and the 5D results (Akpinar *et al*., 2015) support that the polyploidization and domestication of wheat significantly influenced the functional divergence of energy metabolism‐related genes, which could change the productivity and yield of wheat compared to its wild ancestors.

The positively selected genes between Ta7DL and At7DL include TaAP2‐A (TraesCS7D01G178700), FT‐interacting protein (TraesCS7D02G396900) and wall‐associated receptor kinase (TraesCS7D01G545900; Figure 5; Table S13). These genes have already been proven to be associated with yield and grain traits in rice and other crops (Swamy, 2011), indicating they may also be candidate genes involved in wheat domestication and selection. Most of the domestication genes in crops were detected with the characteristic of positive selection and underling traits (Meyer and Purugganan, 2013). Positively selected genes between domesticated wheat and its wild donor were identified. For example, the positively selected GDP‐mannose transporter gene is involved in the synthesis of plant cell surface components such as cell wall polysaccharides (Jing *et al*., 2018). It was reported to be related to carbohydrates and energy, grain yield, grain dry matter content in maize and sorghum (Campbell *et al*., 2016; Fu *et al*., 2010) and grain filling in rice (Rao *et al*., 2011). Grain morphology in wheat has been selected and manipulated even in very early agrarian societies and remains a major breeding target (Gegas *et al*., 2010). Moreover, both the QTL information and GWAS analysis in our study showed that gene loci associated with grain morphology‐related traits were located on a small region around the PSG gene mentioned above (Tables S14, S15). The selection signatures, combined QTLs and GWAS signals, not only provide candidates for functional studies of the domesticated genes involved in important agronomic traits, but also contribute to better understanding the mechanism and patterns of phenotypic evolution in wheat.

## Conclusion

In conclusion, we improved the assembly of the 7DL chromosome arm of bread wheat and then used the high‐quality genomic resource to investigate the sequence, structure and evolution between Ta7DL and At7DL. Our results provide insights into the evolution and genetic consequence of wheat polyploidization, which will accelerate map‐based cloning and support efforts to further improve wheat and the future genome comparative and evolutionary analysis of wheat and related species.

## Experimental procedures

### Physical mapping and sequencing

The 7DL BAC library was constructed from 7DL‐specific DNA discriminated and sorted from flow cytometric analysis of DAPI (4′,6‐diamidino‐2‐phenylindole)‐stained mitotic metaphase chromosomes isolated from double ditelosomic line 7D of cv. Chinese Spring. High molecular weight DNA was prepared from flow‐sorted 7DL arms and used to construct BAC library according to Šimková *et al*. (2008). The 7DL BAC library comprises of 50 304 clones with the average insert size of 116 kb, representing 14.9‐fold coverage of the predicted size of 346 Mb of 7DL (Šimková *et al*., 2011). BAC clones were fingerprinted by HICF SNaPshot (Luo *et al*., 2003) and assembly using the FPC software (Nelson and Soderlund, 2009).

BAC DNA was isolated from BAC clones and sequenced individually using Illumina HiSeq 2500 platform with two insert size libraries of 500 and 800 bp, respectively, following the Illumina's instructions and protocols (Illumina, San Diego, CA). Each BAC clone was isolated for pair‐end library and sequenced individually with 150 bp pair ends and 100 times coverage. A total of 70 Gb of Illumina reads were generated from the MTP clones and singletons. DNA prepared from flow‐sorted 7DL arm was sequenced by Illumina and PacBio technologies, resulting in 26.5 Gb short reads and 3.3 Gb long reads, respectively.

### Sequence assembly and analysis

Each BAC was assembled using SOAP *de novo* (v2.04) separately. In parallel to this effort, Illumina short reads and PacBio long reads of flow‐sorted 7DL arm were used to hybrid assembly of each BAC. Scaffolding BAC sequences and gap filling were facilitated by the physical map together with PacBio reads, sequence derived from singletons and 7DL survey sequence (Berkman *et al*., 2013). After manual sequence elongation and assembly based on overlaps, genetic map information was integrated to construct superscaffolds and a pseudomolecule. A consensus genetic map of 7DL combing several high‐resolution genetic map resources (Saintenac *et al*., 2013; Wang *et al*., 2014) was used to anchor and order the scaffolds and the pseudomolecule.

For the gap close analysis, we used the data of the 3286 manually selected singleton BAC clones, the unmapped sequence of IWGSC V1 version (400 Mb) and the publicly available PacBio sequences (7D chromosome) of cv. Chinese Spring (Clavijo *et al*., 2017). We cut 1500b of the flanking sequence of each (N) region and align them with these three types of data using BLAT (Kent, 2002). To validate the assembly, deletion bin‐mapped ESTs and full‐length cDNA sequences of wheat were used to align against the 7DL draft sequence using BLAT. In order to validate the assembly, four randomly selected MTP clones were sequenced by PacBio platform (100×), and 163 fragments were randomly selected for validation using the Sanger sequencing. Out of them, 149 fragments were successfully sequenced of which 147 were top‐hit matching the assembled sequence of 7DL.

### Annotation of repeated sequences

We combined a homology‐based and *de novo* method to detect repeat sequence in 7DL pseudomolecule sequence. The homologous annotation of repeat sequence was based on searching of the latest TE elements of wheat genome with RepeatMasker (Tarailo‐Graovac and Chen, 2009). In the *de novo* prediction, we firstly constructed a *de novo* repeat library using LTR FINDER (Xu and Wang, 2007) and Piler (Edgar and Myers, 2005). Then, this library was used to identify and classify novel TEs using RepeatMasker. All the repeats were finally combined together with a filtering of those redundant repetitive sequences. The insertion time was counted by the formula of *T* = *K*/2*r*. *T*: element insertion time; *r*: synonymous mutation/site/year; *K*: the divergence between the LTRs and consensus sequence in the TE library.

### Gene prediction and functional analysis

Both homology‐based and transcriptome‐based methods were applied (Jarvis *et al*., 2014) to predict the protein‐coding genes in 7DL. The homologous genes from the orthologous chromosome of related grasses, including *Brachypodium distachyon*,* Oryza sativa*,* Hordeum vulgare* and *S. bicolor*, were aligned to the 7DL genome using TBLASTN (Mount, 2007) with an E‐value cut‐off of 1e^−5^. The genBlastA (She *et al*., 2009) was used to identify the blast hits into candidate gene loci, and GeneWise (Birney *et al*., 2004) was employed to determine gene models to get a final homologous predicted gene set. A total of 39 transcriptome data generated from five tissues of *T. aestivum* cv. Chinese Spring at three different developmental stages (Choulet *et al*., 2014) were used for transcriptome‐based prediction. TopHat (v.2.0; Trapnell *et al*., 2009) was used to align the transcriptome reads against the 7DL assembly, and Cufflinks (Trapnell *et al*., 2014) was used to assemble transcripts using the aligned transcriptome reads. The gene models based on homology‐based annotation and transcriptome‐based prediction were merged to form a comprehensive and nonredundant gene set using GLEAN (Elsik *et al*., 2007). The genes were further functionally annotated by searching against the function database Swissport (Bateman *et al*., 2015), InterProScan (Jones *et al*., 2014) and Nr database (Table S9). Gene Ontology and KEGG (Kanehisa *et al*., 2014) analysis were further performed. Noncoding RNAs in 7DL arms of wheat and *Ae. tauschii* were predicted by using tRNAscan‐SE‐1.23 and infernal of Rfam (Nawrocki *et al*., 2015) software.

### Sequence analysis

Protein sequence data of four species (*Ae. tauschii*,* H. vulgare*,* O. sativa* and *B. distachyon*) were downloaded from Ensembl Plants (plants.ensemble.org/index.html). The protein sequences were combined as a database and performed self‐alignment by all‐to‐all BLASTP (Mount, 2007) with an e‐value of 1e^−5^. The OrthoMCL (Li *et al*., 2003) was used to construct gene family cluster.

Genome data of *Ae. tauschii* were downloaded according to Luo *et al*. (2017). Whole‐genome comparison was performed by using lastz‐1.04.00 software (Harris, 2007) with step of 50 kb. To understand the gene loss events between Ta7DL and At7DL, we used reciprocal BLAST search approach to estimate the numbers of gene difference between them. The orthologs between 7DL arms of wheat *Ae. tauschii* were conducted by InParanoid (Ostlund *et al*., 2010). The alignments were performed using ClustW tool. The dN and dS values were estimated using the ML module integrated in PAML (Yang, 2007). BLAST and BLAT approaches were used to estimate the numbers of gene differences and to find the lost genes. The effective number of codon values, codon adaptation index, codon bias indices and nucleotide contents was investigated using the software CodonW.

### GWAS analysis

The 660K SNP array genotyping of 310 bread wheat accessions is investigated. Then, the grain‐related traits of them, including grain yield, kernel number per spike, grain weight, grain length, grain width, grain diameter, grain colour and spikelet number per spike, were also obtained from crop season 17–18 year. The genotype and phenotype data are available from Cheng *et al*. (2019). GWAS was conducted using TASSEL 5 tools, and the signals with the start and end makers or all the linked markers were obtained. The information of publicly available QTLs in the 7D chromosome was collected, and the linked markers including SSR primers, probe sequence and SNP sites were downloaded from GrainGenes ( https://wheat.pw.usda.gov/GG3/). All the obtained markers were aligned to the pseudomolecule of 7DL by ePCR (Rotmistrovsky *et al*., 2004) and BLASTN.

## Supporting data

The raw sequence data reported in this paper were deposited in the Genome Sequence Archive (2017) in BIG Data Center Members (2017), Beijing Institute of Genomics (BIG), Chinese Academy of Sciences, under accession numbers CRA000647, and are publicly accessible at http://bigd.big.ac.cn/gsa .

## Funding

This work was mainly funded by the National Key Project of Research and Development Program of China (Grant No. 2016YFD0101004 and 2016YFD0100302) and partially supported by the National High‐Tech Research and Development Program (2012AA10A308) from the Chinese Ministry of Science and Technology and special funds for the construction of key disciplines from Northwest A&F University. M.K., H.Š. and J.D. were supported by the Czech Science Foundation (award P501/12/G090) and by the Ministry of Education, Youth and Sports of the Czech Republic (award LO1204 from the National Program of Sustainability I).

## Conflict of interest

The authors declare that there is no conflict of interests.

## Author contributions

S.W., X.N., R.A. and C.Z. designed the project. M.K., H.Š. and J.D. provided 7DL BAC library and amplified chromosomal DNA. X.N., L.W., K.F. and L.C. constructed the physical map. L.C., K.F., D.S., J.J., W.T., X.W., M.W. and H.Z. conducted the genome assembling and predicted gene structure and repeat sequences. X.N., K.F., D.E., D.S. and S.Z. wrote the manuscript. H.D. contributed to data collection and GWAS analysis. M.L., L.M., D.E., R.A., S.H. and X.D. participated in discussions and provided advice. All authors read and approved the final manuscript.

## Supporting information


**Figure S1** Sequence assembly strategy.
**Figure S2** Comparison of MTP clone CS7DL024A18 by *de novo* assembly and PacBio sequencing.
**Figure S3** Evaluation of the assembly by PCR in DNA of Chinese spring.
**Figure S4** Comparison of gene features of wheat 7DL with closely related species (*Hordeum vulgare, Brachypodium distachyon, Oryza sativa,* and *Sorghum bicolor*).
**Figure S5** GO and KEGG classification of genes in 7DL.
**Figure S6** Gene expression patterns of 7DL genes in different tissues and stress conditions.
**Figure S7** Ages of TEs in 7DL.
**Figure S8** Gene order between Ta7DL and At7DL.
**Figure S9** The density of gene loss events along 7DL pseudomolecule compared to *Ae. tauschii*.
**Figure S10** KEGG enrichment of 7DL genes in bread wheat (A) and *Ae. tauschii* (B).
**Figure S11** The frequency distribution of dN, dS and dN/dS in ortholog genes between Ta7DL and At7DL.
**Figure S12** Correlation map of the gene features based on Spearman correlation analysis (positive: red, negative: blue. Insignificant (*P* >= 0.05) value was shown by blank blocks).
**Figure S13** Comparisons of genomic features between positively selected genes (PSGs) and negatively selected genes (NSGs) in 7DL.
**Figure S14** GO classification and KEGG enrichment of PSGs in 7DL.

**Table S1** Assessment of sequence coverage of 7DL assembly by homologous search with deletion‐bin mapped EST sequences
**Table S2** Evaluation of the genome assembly by PacBio sequenced BAC
**Table S3** Summary of non‐coding RNAs
**Table S4** GO annotation of 7DL genes
**Table S5** KEGG annotation of 7DL genes
**Table S6** Transcription factors of 7DL genes
**Table S7** RNA‐Seq data used for gene annotation and expression analysis
**Table S8** FPKM values of 7DL genes in different RNA‐seq data
**Table S9** Annotation of repeat sequences of 7DL
**Table S10** The gap close improved sequence in 7DL
**Table S11** Location of putative QTLs in 7DL pseudomolecule
**Table S12** Information of lost genes in 7DL
**Table S13** Analysis of positively selected genes
**Table S14** Location of positively selected genes and closely related markers
**Table S15** GWAS results of grain traits on 7DL
