# Supplementary material for: The improved assembly of 7DL chromosome provides insight into the structure and evolution of bread wheat
Source: Plant Biotechnol J. 2019 Sep 18;18(3):732–42. doi: 10.1111/pbi.13240 (PMC7004910; doi:10.1111/pbi.13240)
Supplement: Supplementary file 1 — Figure S1 Sequence assembly strategy. Figure S2 Comparison of MTP clone CS7DL024A18 by de novo assembly and PacBio sequencing. Figure S3 Evaluation of the assembly by PCR in DNA of Chinese spring. Figure S4 Comparison of gene features of wheat 7DL with closely related species (Hordeum vulgare, Brachypodium distachyon, Oryza sativa, and Sorghum bicolor). Figure S5 GO and KEGG classification of genes in 7DL. Figure S6 Gene expression patterns of 7DL genes in different tissues and stress conditions. Figure S7 Ages of TEs in 7DL. Figure S8 Gene order between Ta7DL and At7DL. Figure S9 The density of gene loss events along 7DL pseudomolecule compared to Ae. tauschii. Figure S10 KEGG enrichment of 7DL genes in bread wheat (A) and Ae. tauschii (B). Figure S11 The frequency distribution of dN, dS and dN/dS in ortholog genes between Ta7DL and At7DL. Figure S12 Correlation map of the gene features based on Spearman correlation analysis (positive: red, negative: blue. Insignificant (P >= 0.05) value was shown by blank blocks). Figure S13 Comparisons of genomic features between positively selected genes (PSGs) and negatively selected genes (NSGs) in 7DL. Figure S14 GO classification and KEGG enrichment of PSGs in 7DL. [file PBI-18-732-s002.pdf]

Supplemental Figures

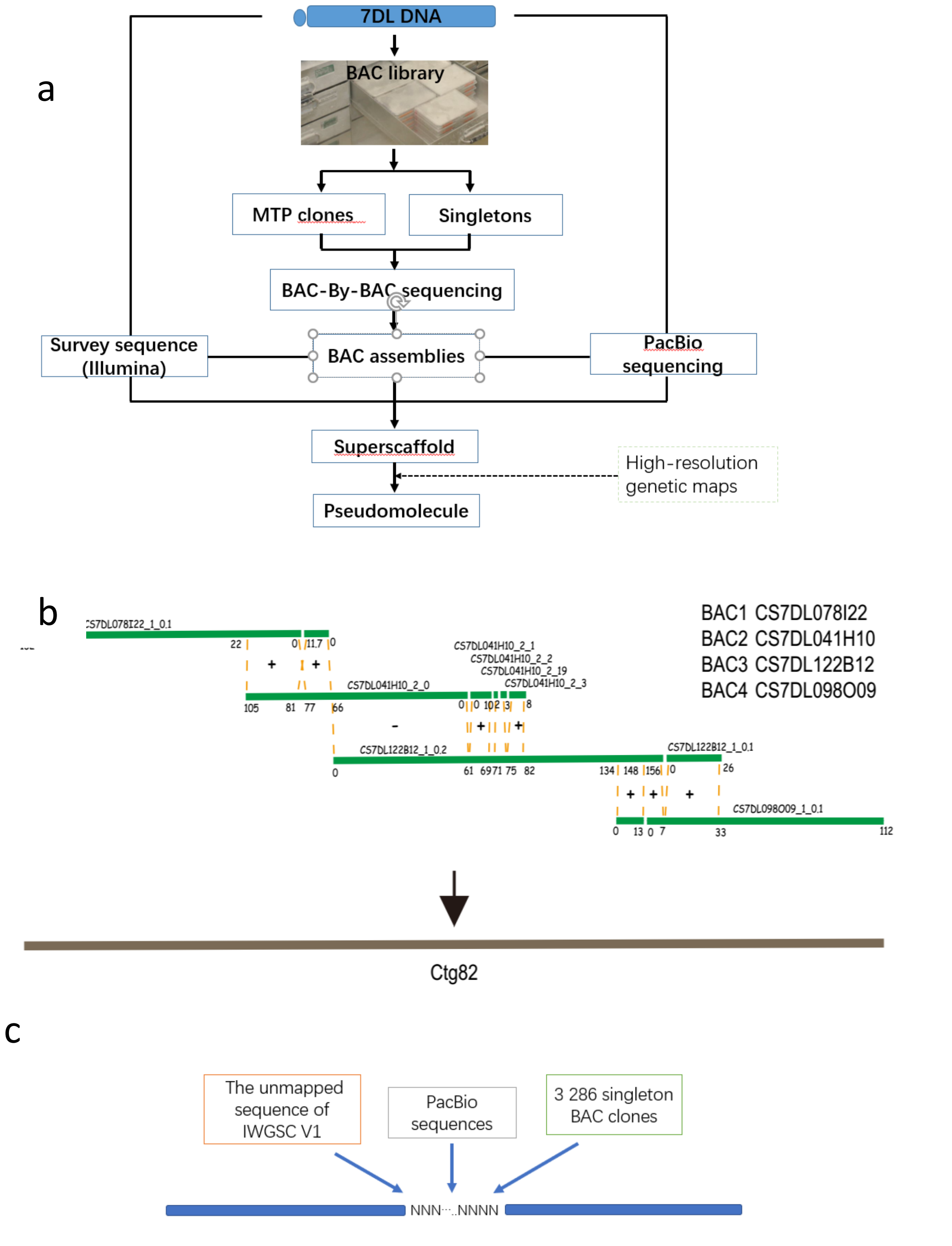

Figure S1: Sequence assembly strategy. (a) Data integration pipeline for the assembly of 7DL. (b) A case of BAC assemblies. (c) The gap closing of 7DL.

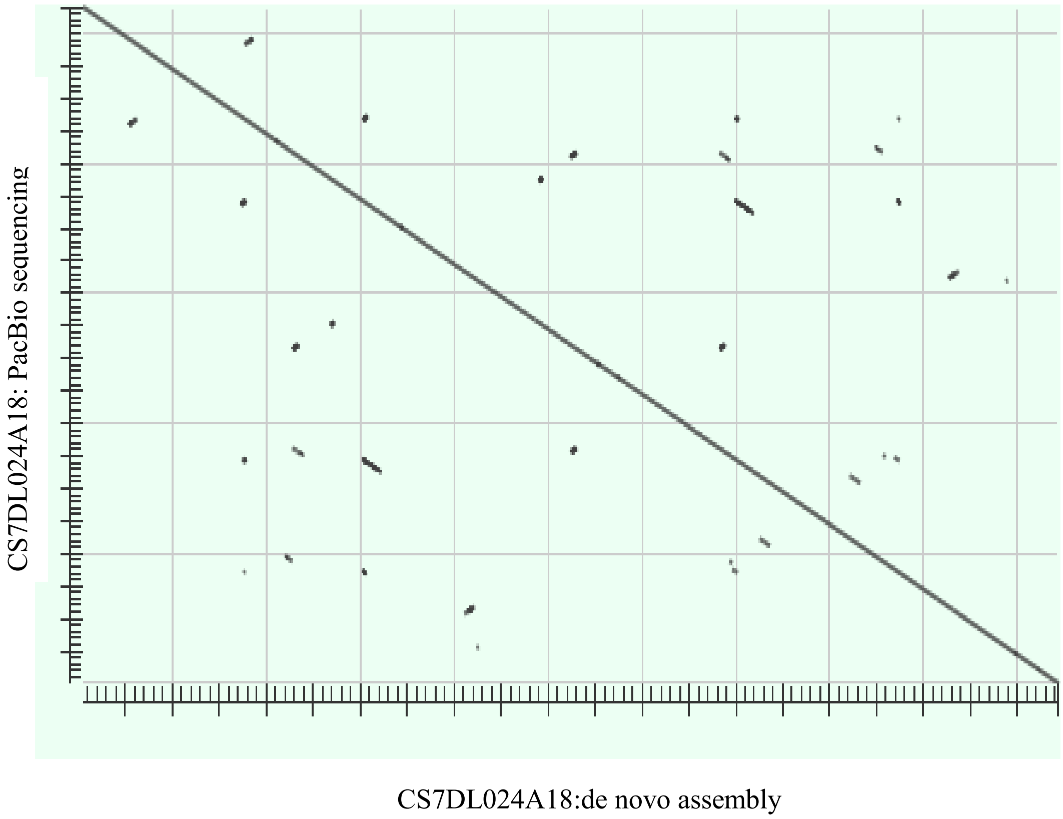

Figure S2. Comparison of MTP clone CS7DL024A18 by de novo assembly and PacBio sequencing

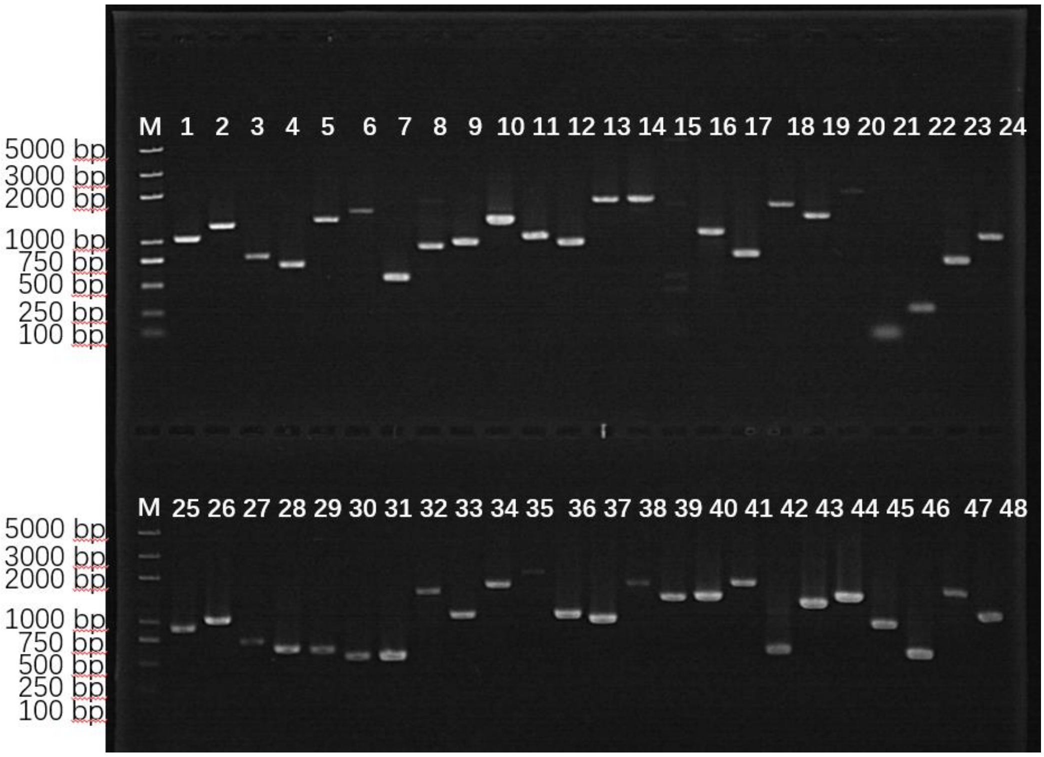

Figure S3. Evaluation of the assembly by PCR in DNA of Chinese spring. (M: DL5000 marker; 1-48: Randomly selected regions in different BACs for PCR validation)

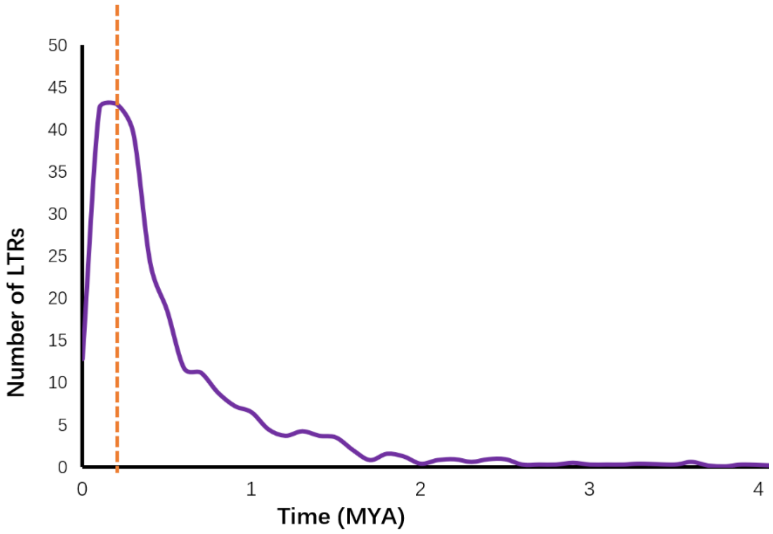

Figure S4. Ages of TEs in 7DL.

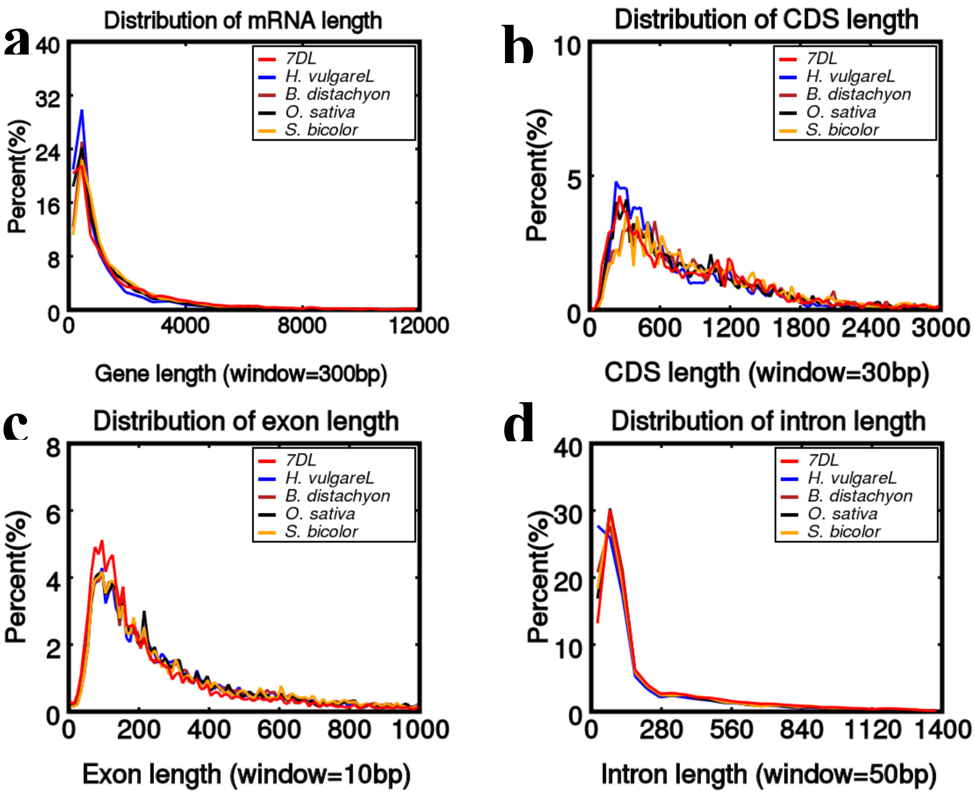

Figure S5. Comparison of gene features of wheat 7DL with closely related species (*Hordeum vulgare, Brachypodium distachyon, Oryza sativa,* and *Sorghum bicolor*)

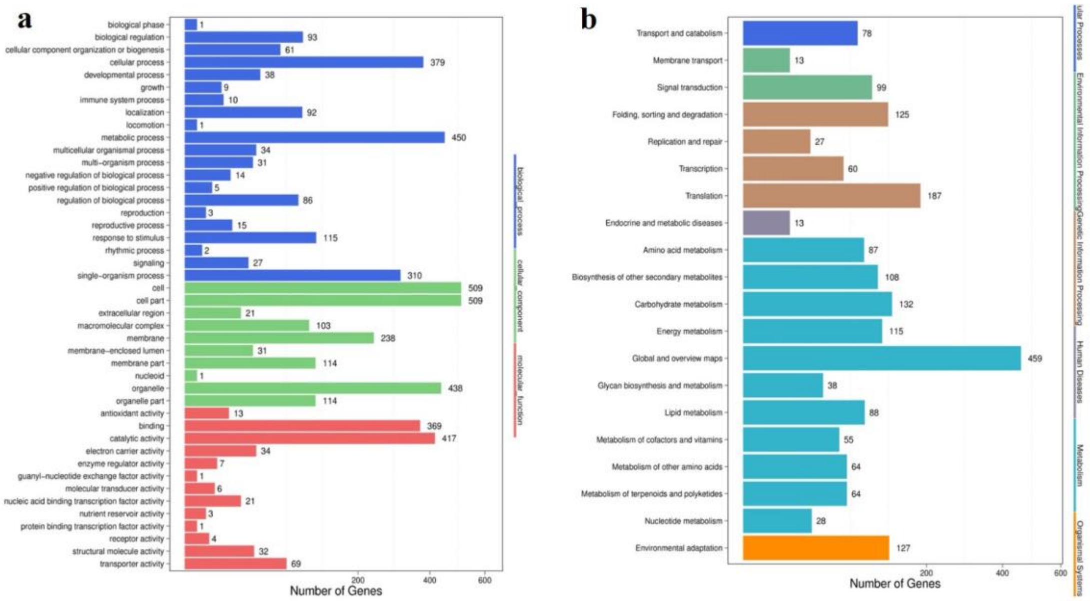

Figure S6. GO and KEGG classification of genes in 7DL

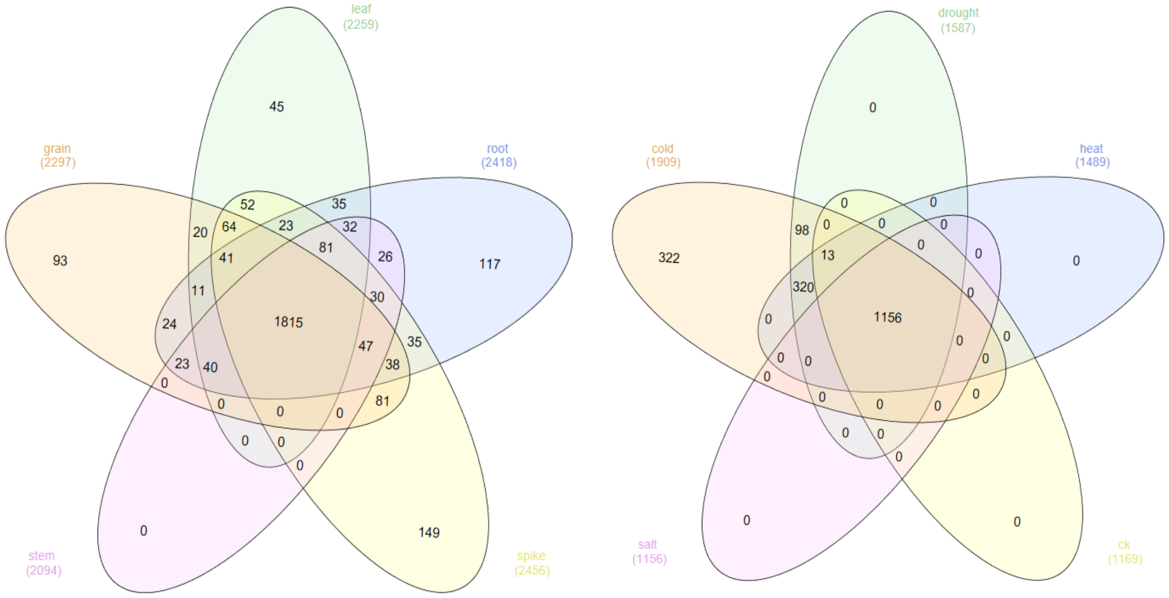

Figure S7. Gene expression patterns of 7DL genes in different tissues and stress conditions. a: Expression patterns in different tissues (leaf, root, spike, stem and grain). b: Expression patterns under different stress conditions (drought, heat, salt, cold and with ck (normal condition).

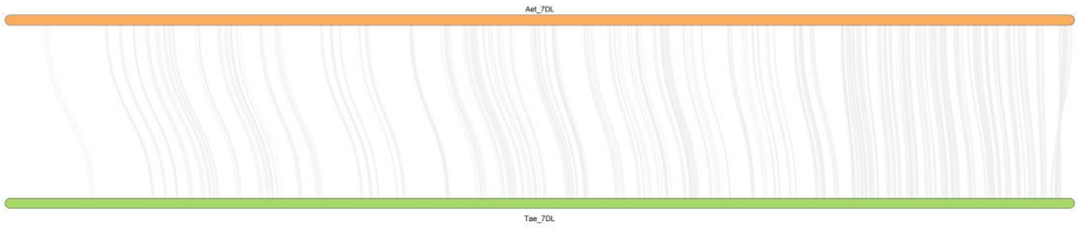

Figure S8. Gene order between Ta7DL and At7DL. (from left to right: centromere to telomere).

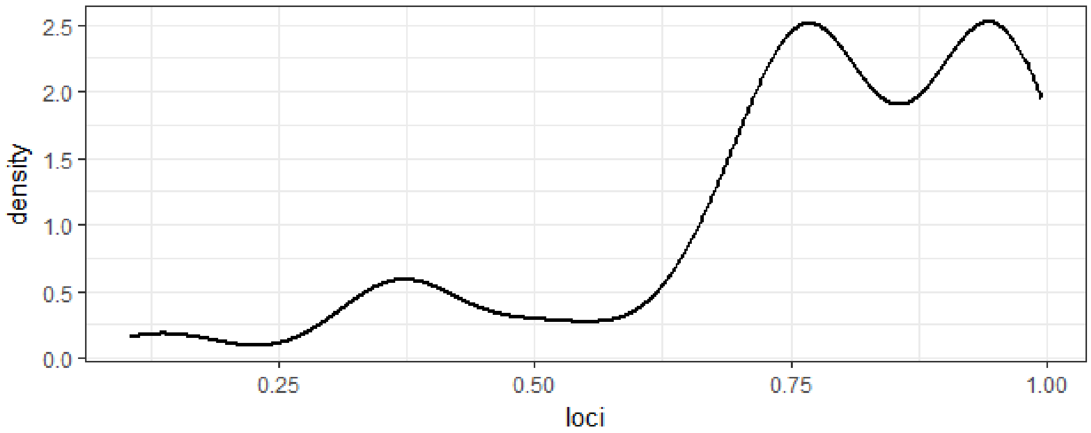

Figure S9. The density of gene loss events along 7DL pseudomolecule compared to *Ae. tauschii.* (horizontal axis: the relative site of 7DL pseudomolecule. from 0 to 1.00 means from centromere to telomere; vertical axis: density of loss genes).

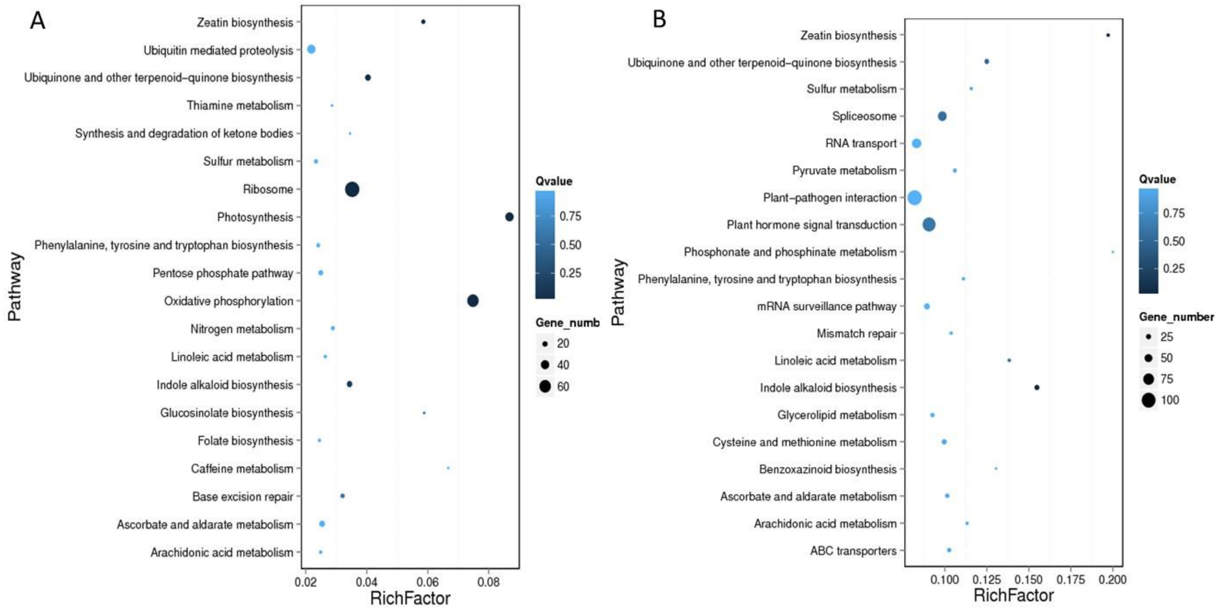

Figure S10. KEGG enrichment of 7DL genes in bread wheat (A) and *Ae. tauschii.* (B)

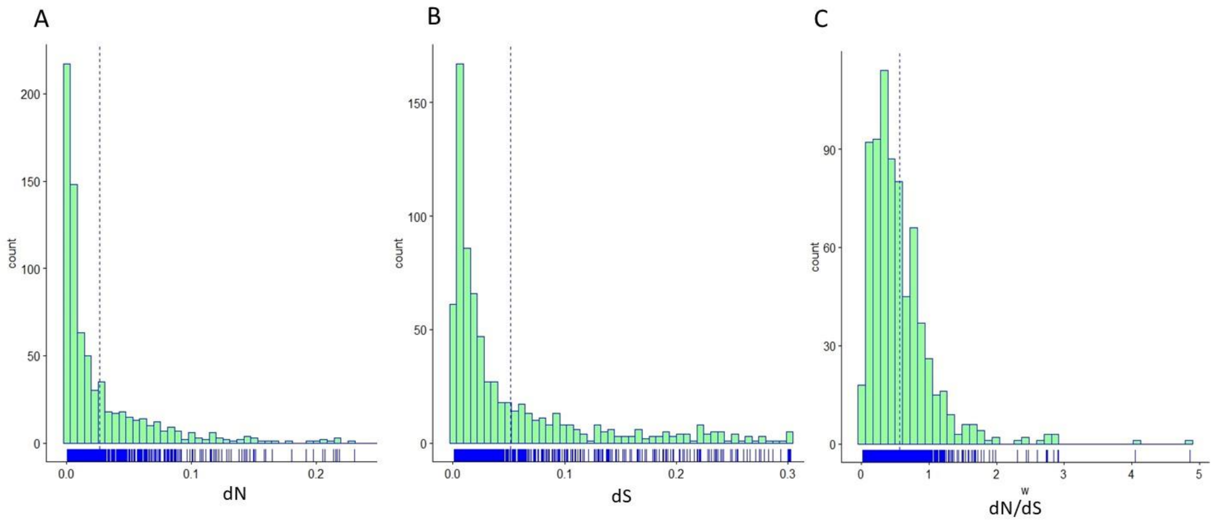

Figure S11. The frequency distribution of dN, dS and dN/dS in ortholog genes between Ta7DL and Ae7DL. (A-C) The frequency displays of dN, dS and dN/dS values, respectively.

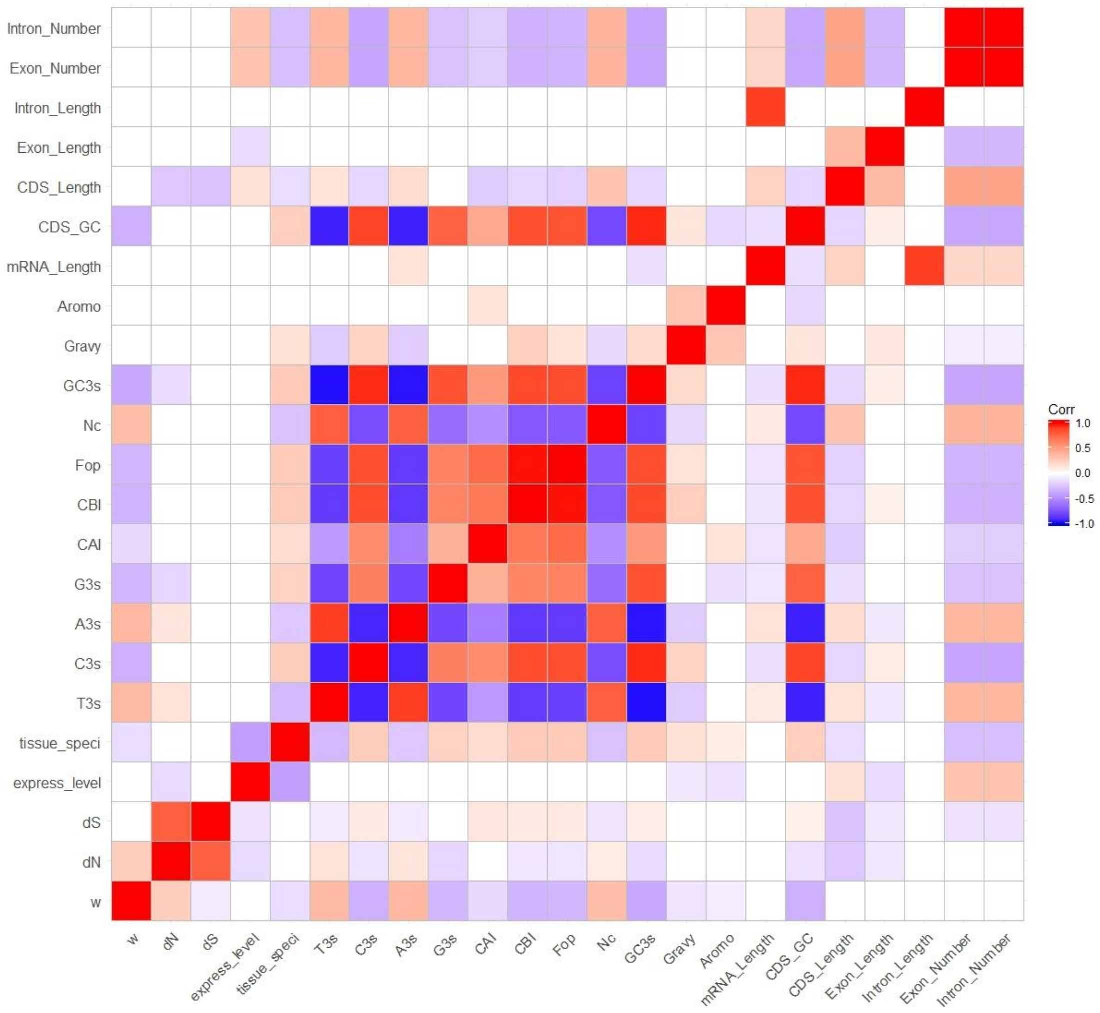

Figure S12. Correlation map of the gene features based on Spearman correlation analysis (positive: red, negative: blue. Insignificant (p >= 0.05) value was shown by blank blocks)

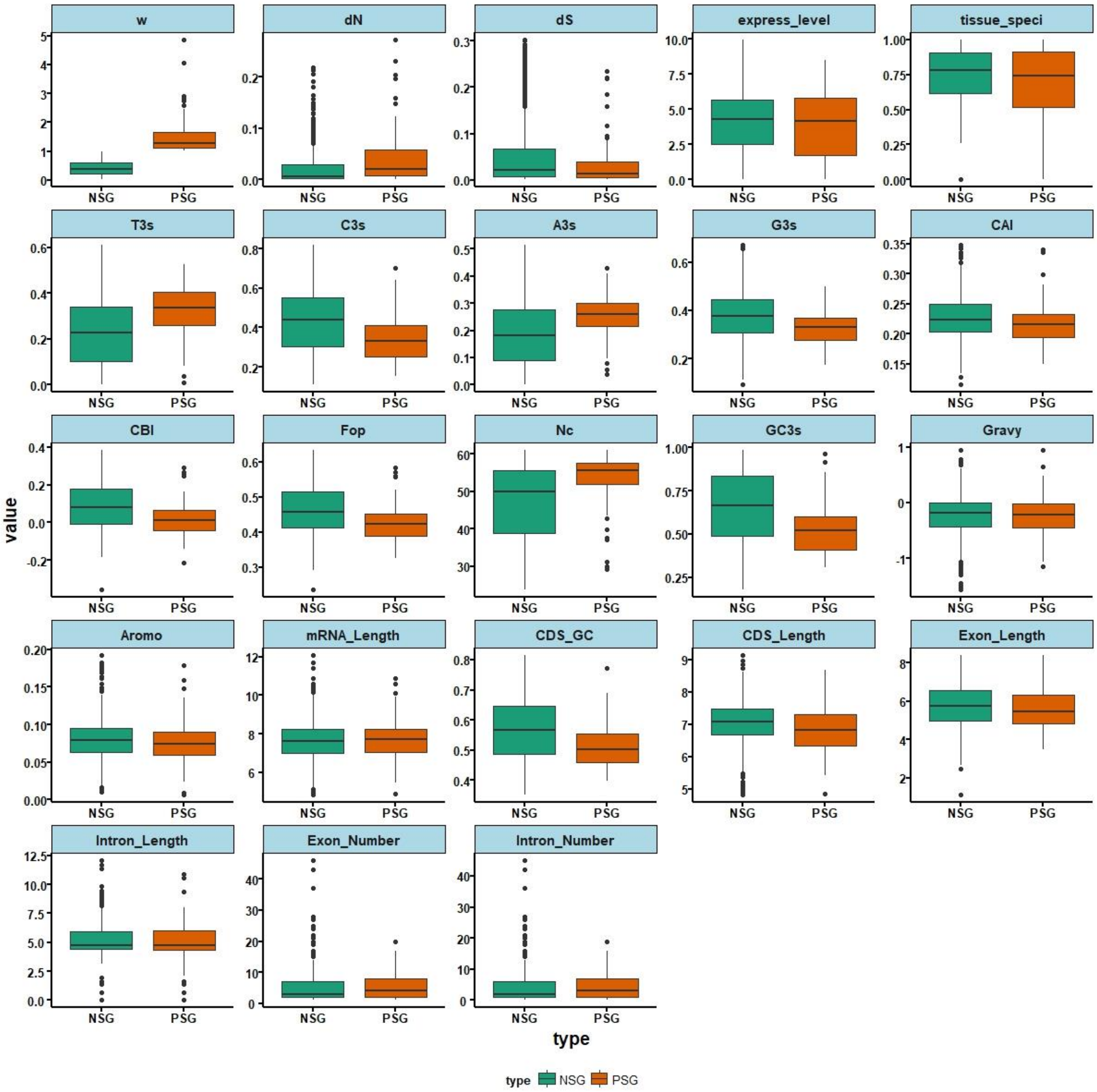

Figure S13. Comparisons of genomic features between positively selected genes (PSGs) and negatively selected genes (NSGs) in 7DL. (Express_level: log(FPKM+1); mRNA_length, CDS_length, Exon_length, Intron_length: log(len+1); len:bp)

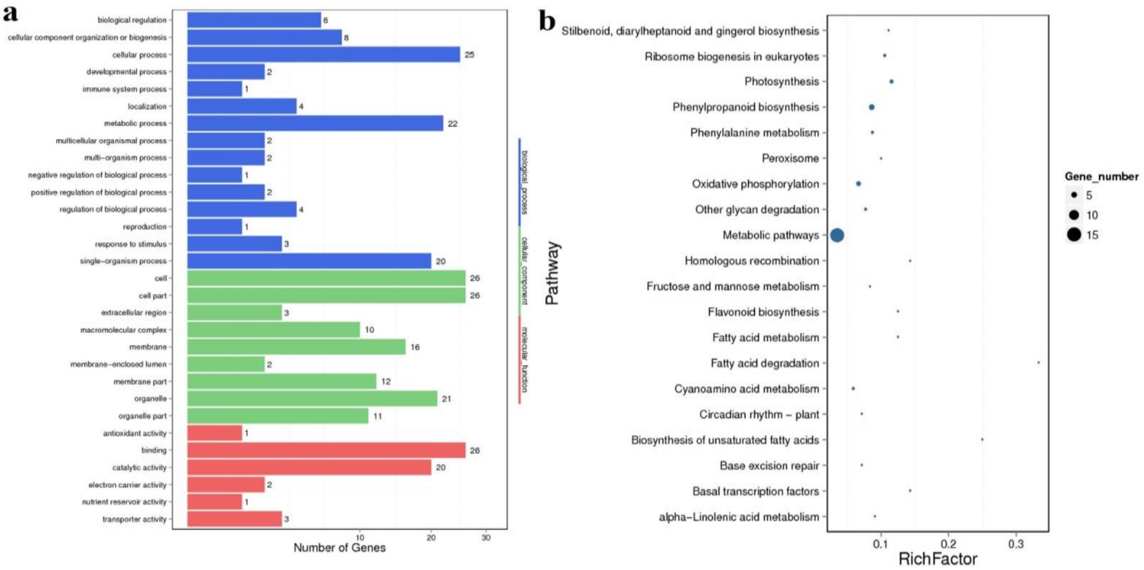

Figure S14. GO classification and KEGG enrichment of PSGs in 7DL
